# 异基因造血干细胞移植治疗NPM1基因突变阳性骨髓增生异常综合征14例临床疗效

**DOI:** 10.3760/cma.j.issn.0253-2727.2023.01.012

**Published:** 2023-01

**Authors:** 依 王, 佳 陈, 胜利 薛, 悦 韩, 晓文 唐, 惠英 仇, 德沛 吴, 荧 王

**Affiliations:** 苏州大学附属第一医院血液内科，江苏省血液研究所，国家血液系统疾病临床医学研究中心，苏州 215006 The First Affiliated Hospital of Soochow University, Jiangsu Institute of Hematology, National Clinical Research Center for Hematologic Diseases, Suzhou 215006, China

NPM1基因突变主要见于急性髓系白血病（AML），但也存在于骨髓增生异常综合征（MDS）和骨髓增殖性肿瘤（MPN）[1]。在MDS患者中，NPM1基因突变率约为4％[2]–[3]。NPM1基因突变阳性MDS患者往往具有原始细胞比例高、病程侵袭和易向AML转化的特点，预后分层多隶属中/高危组，具有异基因造血干细胞移植（allo-HSCT）适应证[1],[4]–[5]。NPM1基因突变AML患者具有独立的预后特征[6]。本研究对近年来在本中心接受allo-HSCT治疗的14例NPM1基因突变阳性MDS患者进行回顾性分析。

## 病例与方法

1. 病例：本研究纳入2015年7月至2020年6月在苏州大学附属第一医院接受allo-HSCT的14例伴NPM1基因突变的MDS患者。所有患者均知情同意。

2. 基因突变检测：2018年前采用一代测序法检测NPM1基因第12号外显子，之后采用二代基因测序技术。

3. 诱导治疗：多数患者allo-HSCT前接受降肿瘤负荷治疗：去甲基化药物（地西他滨）单药或联合半疗程预激方案（阿克拉霉素或去甲氧柔红霉素+阿糖胞苷+G-CSF）。疗效评估参照2019年版MDS中国诊断与治疗指南[5]。

4. 移植方案：所有患者均采用改良白消安/环磷酰胺的清髓性预处理方案。同胞全相合造血干细胞移植患者采用环孢素A联合短程甲氨蝶呤预防移植物抗宿主病，单倍体造血干细胞移植患者在此基础上联合抗胸腺细胞球蛋白（ATG）及霉酚酸酯。移植物为G-CSF动员的骨髓和（或）外周血干细胞。

5. 随访：以门诊及电话方式随访，随访截止日期为2021年9月30日。生存患者中位随访时间47（18～69）个月。

## 结果

1. 一般资料：全部14例患者中男4例，女10例，移植时中位年龄38（16～54）岁。所有患者均隶属MDS伴原始细胞增多型（MDS-EB），其中EB-1患者4例，EB-2患者10例，中位IPSS-R评分5（3.5～7）分。14例患者中，A型突变11例，D型、H型和罕见型各1例。12例患者伴有其他基因突变，其中FLT3-ITD 4例，RAS 4例，DNMT3A 3例。12例患者有染色体核型分析结果，其中11例为正常核型，1例为21号染色体四体（+21,+21）。患者具体临床资料见表1。

**表1:** 14例NPM1基因突变阳性骨髓增生异常综合征患者一般资料及移植前治疗

例号	性别	年龄（岁）	诊断	IPSS-R评分	染色体核型	突变类型	合并突变	移植前治疗方案	疗效
1	女	28	EB-1	6	正常	A型	无	BSC	/
2	女	38	EB-1	5	正常	H型	FLT3-ITD/CEBPA-TAD1	BSC	/
3	男	16	EB-1	3.5	-	罕见型	TET2/WT1/FLT3/ETV6	BSC	/
4	男	41	EB-1	4.5	正常	A型	DNMT3A/FLT3-ITD	地西他滨+CAG	CR
5	女	24	EB-2	7	正常	A型	无	地西他滨+CAG，地西他滨+IAG	CR
6	女	40	EB-2	6.5	正常	A型	CBL/DNMT3A/GATA2	地西他滨+半量CAG	CR
7	女	29	EB-2	6.5	正常	A型	DNMT3A/FLT3-ITD	地西他滨+IAG	mCR
8	男	23	EB-2	5.5	-	A型	BCOR	BSC	/
9	女	38	EB-2	5	正常	D型	NRAS/JAK2	地西他滨+CAG	CR
10	女	38	EB-2	7	正常	A型	KRAS/BCOR/FBXW7/NRAS	地西他滨+IAG	CR
11	女	39	EB-2	5	正常	A型	FLT3-ITD	FLAG	CR
12	女	50	EB-2	4.5	正常	A型	IDH2/KRAS	地西他滨+CAG	CR
13	男	20	EB-2	5	正常	A型	IDH1	地西他滨	SD
14	女	54	EB-2	5.5	48,XX,+21,+21	A型	NRAS	地西他滨+IAG	CR

注 IPSS-R：骨髓增生异常综合征修订国际预后积分系统；EB-1：骨髓增生异常综合征伴原始细胞增多1型；BSC：最佳支持治疗；CAG：阿克拉霉素+阿糖胞苷+粒细胞集落刺激因子；IAG：去甲氧柔红霉素+阿糖胞苷+粒细胞集落刺激因子；FLAG：氟达拉滨+阿糖胞苷+粒细胞集落刺激因子；CR：完全缓解；mCR：骨髓完全缓解；SD：疾病稳定；-：未见分裂象；/：不适用

2. 移植前治疗：10例MDS-EB2患者中，7例移植前接受地西他滨联合预激方案治疗，1例患者接受FLAG方案诱导治疗，7例患者获得完全缓解（CR），1例获得骨髓完全缓解（mCR）。4例MDS-EB1患者中，1例接受地西他滨联合预激方案诱导，获得CR。诱导治疗过程中患者耐受良好，不良反应多为Ⅰ/Ⅱ级。所有患者化疗后均发生Ⅲ/Ⅳ级骨髓抑制，中性粒细胞绝对计数（ANC）<0.5×10^9^/L、血小板计数<20×10^9^/L的中位持续时间分别为23（0～32）d、25（19～31）d。5例患者出现粒细胞缺乏伴发热（感染部位不明确），1例患者发生肺部感染。

3. 移植情况：3例患者行同胞全相合造血干细胞移植，11例行单倍体造血干细胞移植。例4于移植后10个月复发，移植后14个月死于本病。例6移植后21 d粒系重建，但移植后26 d死于脑出血；例12植入失败，二次移植预处理期间死于脑出血；其余11例患者均获得完全植入且无病生存至今，中位存活时间为44（1～69）个月。全部14例患者的移植情况见表2。

**表2:** 14例NPM1基因突变阳性骨髓增生异常综合征患者移植资料

例号	移植类型	移植物	预处理	GVHD预防	回输MNC（×10^8^/kg）	回输CD34^+^细胞（×10^6^/kg）	粒细胞植入时间（d）	血小板植入时间（d）	随访及转归
1	sib-HSCT	BM+PB	BU/CY	CsA+MTX	14.19	6.95	10	11	移植后69个月，无病生存
2	sib-HSCT	PB	BU/CY	CsA+MTX	6.73	3.32	11	11	移植后61个月，无病生存
3	haplo-HSCT	BM+PB	BU/CY+ATG	CsA+MTX+MMF	7.37	2.83	12	19	移植后46个月，无病生存
4	haplo-HSCT	BM+PB	BU/CY+ATG	CsA+MTX+MMF	13.50	3.28	12	32	移植后10个月复发，14个月死亡
5	haplo-HSCT	BM+PB	BU/CY+ATG	CsA+MTX+MMF	14.12	3.77	11	11	移植后69个月，无病生存
6	haplo-HSCT	BM	BU/CY+ATG	CsA+MTX+MMF	8.70	5.05	21	–	移植后26 d死于脑出血
7	haplo-HSCT	BM+PB	BU/CY+ATG	CsA+MTX+MMF	16.39	3.13	13	13	移植后52个月，无病生存
8	haplo-HSCT	BM+PB	BU/CY+ATG	CsA+MTX+MMF	9.66	2.89	14	18	移植后50个月，无病生存
9	sib-HSCT	PB	BU/CY	CsA+MTX	11.70	3.74	12	13	移植后47个月，无病生存
10	haplo-HSCT	BM+PB	BU/CY+ATG	CsA+MTX+MMF	8.87	10.22	11	11	移植后42个月，无病生存
11	haplo-HSCT	BM+PB	BU/CY+ATG	CsA+MTX+MMF	12.10	5.07	12	10	移植后33个月，无病生存
12	haplo-HSCT	BM+PB	BU/CY+ATG	CsA+MTX+MMF	6.88	3.05	–	–	植入失败，移植后57 d死于脑出血
13	haplo-HSCT	BM+PB	BU/CY+ATG	CsA+MTX+MMF	7.94	3.85	13	14	移植后25个月，无病生存
14	haplo-HSCT	PB	BU/CY+ATG	CsA+MTX+MMF	13.70	5.48	10	11	移植后18个月，无病生存

注 sib-HSCT：同胞全相合造血干细胞移植；haplo-HSCT：单倍体造血干细胞移植；PB：外周血；BM：骨髓；BU：白消安；CY：环磷酰胺；ATG：抗胸腺细胞球蛋白；GVHD：移植物抗宿主病；CsA：环孢素A；MTX：甲氨蝶呤；MMF：霉酚酸酯；MNC：单个核细胞；–：未植入

## 讨论

NPM1基因突变是AML常见的分子学异常，因NPM1突变的AML患者具有独特的临床特征，2016版WHO造血和淋巴组织肿瘤分类已正式将其列为具有重现性遗传学异常AML中的一个独立亚型[6]。NPM1突变也偶见于MDS和MPN[1]–[4]。研究显示，NPM1突变阳性的MDS和MPN患者生物学特征与阴性患者明显不同，绝大多数患者为EB亚型，治疗反应类似于AML患者[1],[4]。根据2043例MDS患者的染色体和基因突变结果，Bersanelli等[7]将MDS划分为8个亚组，其中具有NPM1以及DNMT3A、FLT3、IDH1和RUNX1基因突变的患者定义为“伴AML样基因突变”的第7亚组，组内83％的患者骨髓原始细胞比例15％～19％。本研究中，患者骨髓原始细胞比例较高，女性患者多见，主要见于正常核型，这些特点与NPM1突变AML相似，也体现了这一特性。有学者建议[8]，为便于开展临床研究，应不再以骨髓原始细胞占比（20％）作为区分诊断的界值，而将患者统一归纳为一组名为“NPM1突变的髓系肿瘤”。但这样的分类可能忽视了NPM1突变MDS患者的异质性，少部分NPM1突变MDS患者病情可以相对温和。国内李璘等[2]报道了232例MDS患者的NPM1基因突变检测数据，结果9例（3.9％）患者阳性，其中7例患者为低危组，长期随访结果显示4例患者病情无进展。因此，NPM1基因突变MDS患者的总体临床特征仍需大样本研究才能明确，但有理由相信，原始细胞比例增高和（或）伴有高危基因突变的患者，临床特征可能更接近原发性AML。

Montalban-Bravo等[4]对28例NPM1突变阳性的MDS患者进行了疗效分析，其中10例接受传统化疗的患者9例获得CR，1例获得mCR，CR率显著高于单用去甲基化药物治疗的患者（90％对28％，*P*＝0.004）。本研究中，8例接受地西他滨联合预激方案诱导治疗的患者均获得满意疗效（7例CR，1例mCR），提示这一相对温和的联合方案可以获得与传统高强度化疗一致的疗效。近期临床研究结果显示，阿扎胞苷联合维奈克拉治疗NPM1突变阳性AML患者疗效较好，一线治疗患者复合缓解率超过90％[9]。这一新型去化疗方案对于MDS患者，包括NPM1突变阳性患者的疗效及安全性如何，仍有待数据积累。此外，一般认为，NPM1突变阳性AML患者预后相对良好，尤其是不伴高频FLT3-ITD突变的患者。对于这类预后良好型患者，指南推荐缓解后以大剂量阿糖胞苷作为巩固治疗，复发后再考虑allo-HSCT[6]。但对于NPM1突变阳性的MDS-EB患者，尤其是不伴有其他高危基因突变的患者，是否有可能通过化疗和（或）自体HSCT获得临床治愈，目前尚无大样本临床数据，需要将来进一步研究。

allo-HSCT是目前唯一可能治愈MDS的治疗手段[10]。对于骨髓原始细胞比例>10％的患者，共识建议等待移植期间可应用降肿瘤负荷治疗以降低移植后复发率，提高移植总体疗效，但这一策略尚缺乏前瞻性研究数据支持[11]–[12]。数项回顾性研究提示，移植前接受化疗或去甲基化药物治疗的患者，与直接移植的患者相比，allo-HSCT后OS率无明显差异[13]–[15]，原因猜测主要可能与桥接治疗克隆筛选和复发时克隆演变有关[16]–[17]。但另一方面，尽管可能差异无统计学意义，移植前获得CR的患者，移植后无复发生存率一般高于对照组[13]–[15]。本组患者中，仅有1例患者移植后复发，发生率似低于高危MDS患者allo-HSCT后30％左右的累积复发率[13]–[14]。鉴于CR患者移植耐受性好，移植早期治疗风险相对较低，以及前述的NPM1突变阳性MDS患者临床特征的特殊性，有理由推测，NPM1突变阳性的MDS患者可能从移植前的桥接化疗中获益。

综上所述，本组病例资料显示，NPM1突变阳性MDS-EB患者allo-HSCT前诱导治疗反应率高，allo-HSCT后复发率低，有较好的长期疗效，但本研究入组例数较少，确切结论尚需大样本的研究进一步论证。

## References

[1] Patel SS, Ho C, Ptashkin RN (2019). Clinicopathologic and genetic characterization of nonacute NPM1-mutated myeloid neoplasms[J]. Blood Adv.

[2] 李 璘, 张 悦, 马 晓瑭 (2010). 原发性骨髓增生异常综合征患者NPM1基因突变的研究[J]. 中华血液学杂志.

[3] Bains A, Luthra R, Medeiros LJ (2011). FLT3 and NPM1 mutations in myelodysplastic syndromes: Frequency and potential value for predicting progression to acute myeloid leukemia[J]. Am J Clin Pathol.

[4] Montalban-Bravo G, Kanagal-Shamanna R, Sasaki K (2019). NPM1 mutations define a specific subgroup of MDS and MDS/MPN patients with favorable outcomes with intensive chemotherapy[J]. Blood Adv.

[5] 中华医学会血液学分会 (2019). 骨髓增生异常综合征中国诊断与治疗指南(2019年版)[J]. 中华血液学杂志.

[6] Falini B, Sciabolacci S, Falini L (2021). Diagnostic and therapeutic pitfalls in NPM1-mutated AML: notes from the field[J]. Leukemia.

[7] Bersanelli M, Travaglino E, Meggendorfer M (2021). Classification and personalized prognostic assessment on the basis of clinical and genomic features in myelodysplastic syndromes[J]. J Clin Oncol.

[8] Estey E, Hasserjian RP, Döhner H (2022). Distinguishing AML from MDS: a fixed blast percentage may no longer be optimal[J]. Blood.

[9] Lachowiez CA, Loghavi S, Kadia TM (2020). Outcomes of older patients with NPM1-mutated AML: current treatments and the promise of venetoclax-based regimens[J]. Blood Adv.

[10] Cazzola M (2020). Myelodysplastic Syndromes[J]. N Engl J Med.

[11] Garcia-Manero G, Chien KS, Montalban-Bravo G (2020). Myelodysplastic syndromes: 2021 update on diagnosis, risk stratification and management[J]. Am J Hematol.

[12] Malcovati L, Hellström-Lindberg E, Bowen D (2013). Diagnosis and treatment of primary myelodysplastic syndromes in adults: recommendations from the European LeukemiaNet[J]. Blood.

[13] Damaj G, Duhamel A, Robin M (2012). Impact of azacitidine before allogeneic stem-cell transplantation for myelodysplastic syndromes: a study by the Societe Francaise de Greffe de Moelle et de Therapie-Cellulaire and the Groupe-Francophone des Myelodysplasies[J]. J Clin Oncol.

[14] Schroeder T, Wegener N, Lauseker M (2019). Comparison between upfront transplantation and different pretransplant cytoreductive treatment approaches in patients with high-risk myelodysplastic syndrome and secondary acute myelogenous leukemia[J]. Biol Blood Marrow Transplant.

[15] Qin Y, Kuang P, Zeng Q (2019). Hypomethylating agents for patients with myelodysplastic syndromes prior to hematopoietic stem cell transplantation: a systematic review and meta-analysis[J]. Ann Hematol.

[16] da Silva-Coelho P, Kroeze LI, Yoshida K (2017). Clonal evolution in myelodysplastic syndromes[J]. Nat Commun.

[17] Jacoby MA, Duncavage EJ, Chang GS (2018). Subclones dominate at MDS progression following allogeneic hematopoietic cell transplant[J]. JCI Insight.

